# The Development and Progression of Micro-Nano Optics

**DOI:** 10.3389/fchem.2022.916553

**Published:** 2022-06-20

**Authors:** Yong Wang, Jie Yang, Zhiwei Wang, Xiaofei Kong, Xiangyu Sun, Jingjing Tian, Xiushuo Zhang, Xiaolong Zhao, Yanping Liu, Hongsheng Li, Yuqing Su, Xiaorui Hao, Jing Xu

**Affiliations:** ^1^ Laboratory of Optical Detection and Imaging, School of Science, Qingdao University of Technology, Qingdao, China; ^2^ Quantum Physics Laboratory, School of Science, Qingdao University of Technology, Qingdao, China; ^3^ Qingdao Technology Innovation Center of Remote Sensing and Precise Measurement, Qingdao, China; ^4^ Torch High Technology Industry Development Center, Ministry of Science and Technology, Beijing, China

**Keywords:** micro-nano optics, luminescent materials, optical waveguides, photoelectric detection, structures, review

## Abstract

Micro-Nano optics is one of the most active frontiers in the current development of optics. It combines the cutting-edge achievements of photonics and nanotechnology, which can realize many brand-new functions on the basis of local electromagnetic interactions and become an indispensable key science and technology of the 21st century. Micro-Nano optics is also an important development direction of the new optoelectronics industry at present. It plays an irreplaceable role in optical communication, optical interconnection, optical storage, sensing imaging, sensing measurement, display, solid-state lighting, biomedicine, security, green energy, and other fields. In this paper, we will summarize the research status of micro-nano optics, and analyze it from four aspects: micro-nano luminescent materials and devices, micro-nano optical waveguide materials and devices, micro-nano photoelectric detection materials and devices, and micro-nano optical structures and devices. Finally, the future development of micro-nano optics will be prospected.

## Introduction

Micro-nano optics, also known as micro-optics, is an emerging research direction resulting from the organic combination of optics and nanotechnology, which carries the scientific extension of traditional optics at sub-wavelength scales and promotes the development of substantive innovations in nanotechnology with the help of optical platforms. It is an optical theory for the study of micro-nano optical structures and the study of micro-optical components, systems, and devices on the micro-nano scale. Micro-nano optics is not only one of the important development directions of the optoelectronics industry, but also a frontier research direction in the field of optics, which has a wide range of application prospects in many fields such as optical communication, optical storage, laser nuclear fusion engineering, laser weapons, solar energy utilization, semiconductor laser, and optical anti-counterfeiting technology, etc. It is the frontier of current optical research and an important branch. In 1852, Faraday first reported on the experimentally detected optical properties of metal nanoparticles (Faraday, 1857). As early as 1880, before the concept of “integrated optics” was proposed, Wheeler first mentioned a similar concept of optical waveguides in a patent, calling it an “optical pipe” (Wheeler, 1881). The structure is similar to the well-known optical fiber structure, which can realize point-to-point light wave transmission. The study of dielectric optical waveguides began in 1910 when Hondros and Debye (1910) proposed the theory of dielectric waveguides based on the study of cylindrical dielectric optical waveguides. In 1936, Carson et al. (1936) theoretically refined the dielectric guided-mode transmission, and in the same year, Southworth (1936) started a related experimental study. In 1964, Osterberg and Smith (1964) completed the first experiments on beam coupling in planar waveguides using prisms and thin slides to make a theoretical analysis of optical field coupling. In the same year, Schlosser (1964) conducted a theoretical study on rectangular waveguides. In 1965, Anderson (1965) developed a thin-film planar optical waveguide that could be used for transmission in the infrared band. Based on the above-mentioned extensive research work, the requirements for optical waveguides have gradually increased, and are not only limited to the field of low-loss and long-range transmission of optical fiber, but also multi-functional integrated optical waveguide devices have attracted extensive attention from scientists since the beginning of the 1969. In 1960, the world’s first ruby solid-state laser was introduced (Maiman, 2018). Compared to electrical signals such as radio and microwave, this monochromatic coherent light generation provides a stable high frequency signal carrier and solves the problem of information transmission bandwidth. In order to avoid the influence of atmospheric or climatic conditions when the light signal propagates in the air medium, people began to try to use various optical materials as the transmission medium of light to reduce transmission loss. In 1966, the scientist Gao set up the idea that light signals could be transmitted in pure glass-based optical fibers with low loss, ushering in a new era of optical fiber communication (Kao and Hockham, 1966). In 1969, Miller of Bell Labs first introduced the concept of “integrated optics,” which is similar to “integrated circuits” (Hunsperger, 2009). By reducing the size of traditional optical components, optical devices with different functions, such as light sources, optical waveguides and optical detectors, are integrated into photonic chips to achieve a variety of functions such as information processing and information transmission. Compared with integrated circuits, the transmission speed and frequency of light waves are high, and the linear transmission of light waves of different wavelengths in optical media does not interfere with each other, so integrated optical systems based on optical “carrier” signals have greater information capacity, wider transmission bandwidth, faster transmission speed, lower transmission loss, and more stable transmission performance (Tamir, 1975; Ginés, 2003; Kokubun and Iga, 2005; Okamoto, 2006; Bohren and Huffman, 2008; Olson et al., 2014). Compared to conventional optical systems, integrated optical systems are not only smaller and lighter, but also more efficient and stable, which is why they are favored by scientists.

In 1987, Yabnolovitch (1987a) and John (1987) extended the concept of electron energy bands to light waves when discussing how to suppress spontaneous radiation from atoms and photonic localization, introducing the concept of photonic crystals. In 1998, Ebbesen et al. (1998) discovered the existence of super-intense light transmission peaks in thick metallic films punched with periodic subwavelength nanopores, a discovery that sparked a wave of research into surface equipartition excitations in metallic periodic structures. Since 1987, the research of micro-nano optical structure has been developing vigorously in various fields.

## Current Status of Research in Micro-Nano Optics

This paper will summarize the development of micro-nano optics in four directions: micro-nano emitting materials and devices, micro-nano optical waveguide materials and devices, micro-nano optical detection materials and devices, and micro-nano optical structures and devices.

### Micro-Nano Luminescent Materials and Devices

**FIGURE 1:**
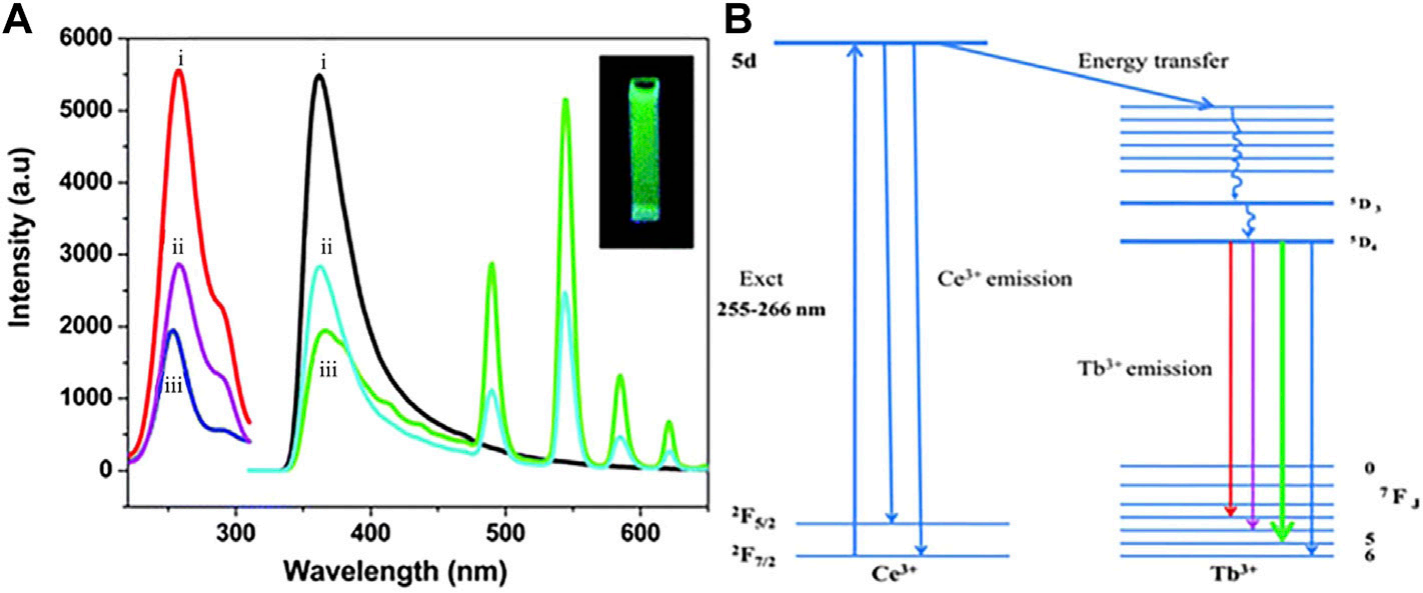
**(A)** Excitation and emission spectra. 1) 1 mol% Ce^3+^ doped, 2) 1 mol% Ce^3+^ and 0.5 mol% Tb^3+^ doped, 3) 1 mol% Ce^3+^ doped and 0.5 mol% Tb^3+^ coated Na(Y1.5Na0.5)F6 nanocrystals. **(B)** Energy level diagram for electronic transitions and energy transfer process of Na(Y1.5Na0.5)F6:Ce:Tb (Ghosh et al., 2010).

**FIGURE 2:**
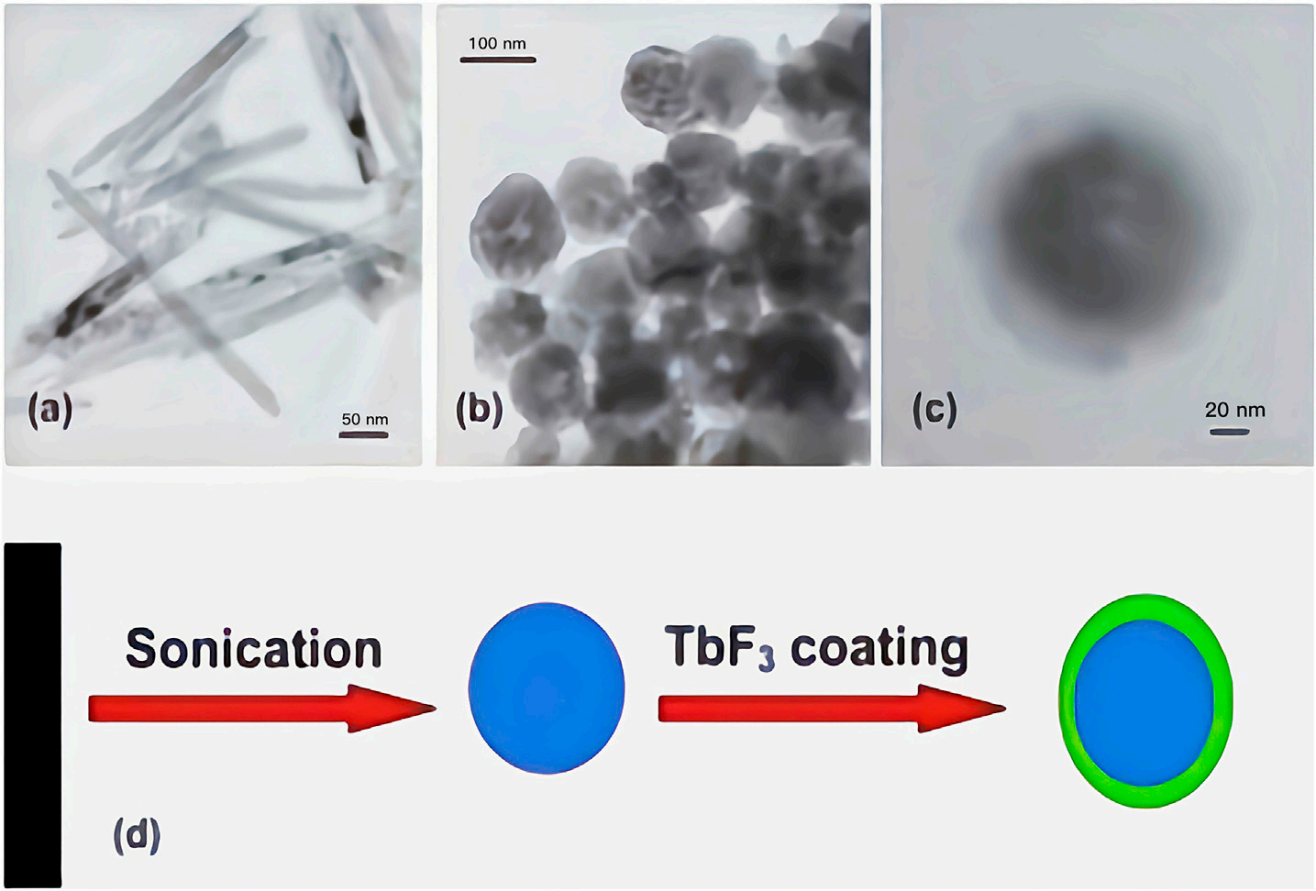
Morphology of Na(Y1.5Na0.5)F6:Ce. **(A)** Low magnification TEM images of 75 C dried Na(Y1.5Na0.5)F6:Ce (1) doped nanorods, **(B)** 75 C dried sonicated Na(Y1.5Na0.5)F6:Ce (1) doped nanoparticles, **(C)** core-shell NaYF4:Ce (1)/Tb nanoparticles. **(D)** Schematic diagram of morphology change (Ghosh et al., 2010).

**FIGURE 3:**
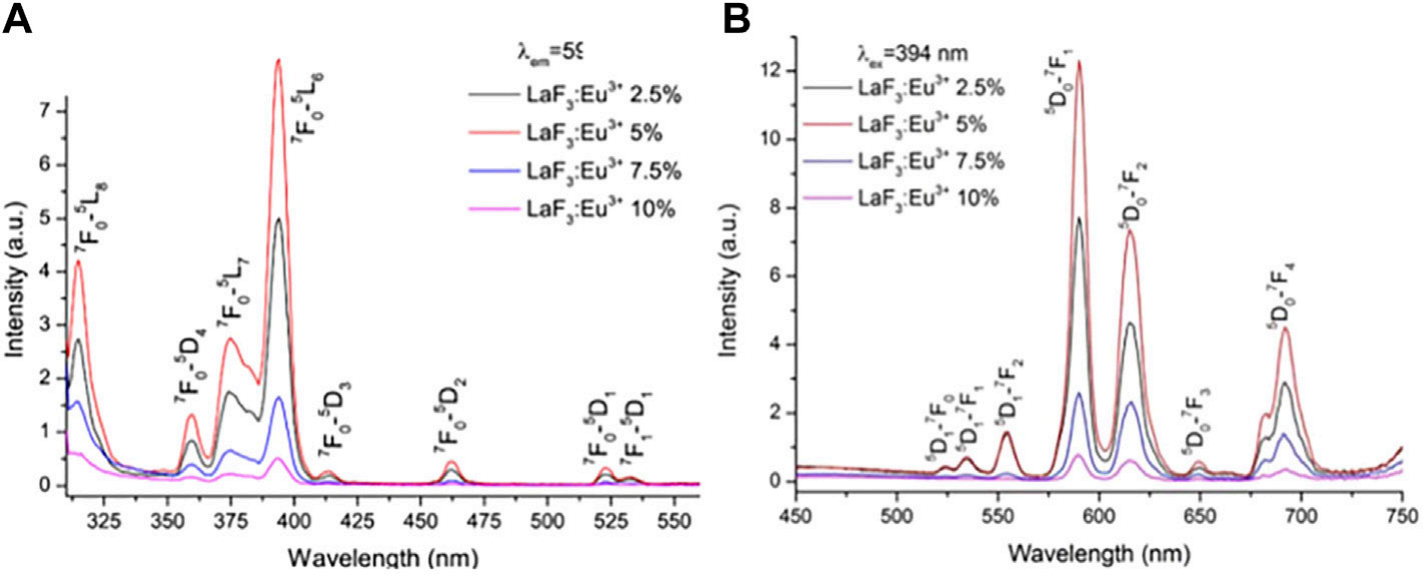
Excitation spectra **(A)** and emission spectra **(B)** of LaF3:Eu^3+^ with different Eu^3+^-doping concentration (Gulina et al., 2017).

**FIGURE 4:**
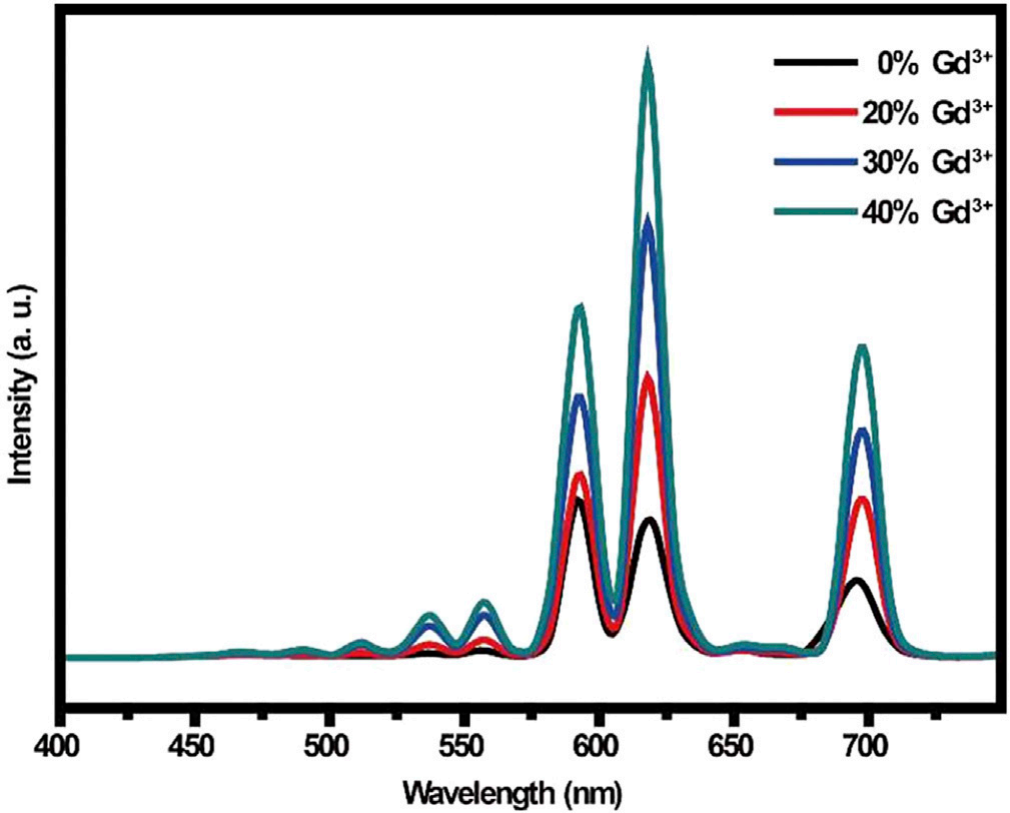
Emission spectra of β-NaLaF4:Eu^3+^, Gd^3+^ red nanomaterials with different Gd^3+^-doping concentration (Nie et al., 2017).

Luminescence is the direct conversion of energy absorbed in some way within an object into non-equilibrium radiation without going through a thermal phase. Luminous materials generally consist of a matrix (the material’s subject compound) and an activator (a small amount of dopant ions that act as a luminous centre). For rare-earth ions co-doped luminescent materials, a sensitizer is usually incorporated into the matrix to absorb the excitation radiation and transfer the energy to the activator. Micro-nano luminescent materials mainly use micro-nano particles as a luminescent matrix, including pure and doped micro-nano semiconductor luminescent materials, rare earth ions and excessive metal ions doped nano-oxides, sulfides, composite oxides, and a variety of micro-nano inorganic salt luminescent materials. For activator materials, there are usually two types of ions: a class of transition metal ions, such as Mn^4+^/Mn^2+^ (Huang et al., 2016a), Ti^4+^ (Page et al., 2010), Cr^3+^ (Chen et al., 2014), Cd^2+^ (Mall and Kumar, 2010), etc.; the second group is rare earth metal ions Eu^3+^/Eu^2+^, and Tb^3+^, Pr^3+^, etc. Fluoride is an ideal matrix material for various fluorescent materials, and the doping of rare earth ions etc. into fluoride matrix can lead to luminescent materials with good optical properties (Kasturi et al., 2016; Keevend et al., 2017). Patra et al. (Ghosh et al., 2010) prepared Na (Y1.5Na0.5) F_6_:Ce:Tb nanomaterials by microemulsion method, and studied their morphological, structure and luminescent properties (see Figure 1). They found that Na (Y1.5Na0.5) F_6_:Ce:Tb nanorods or nanowires could be prepared by using cationic surfactant cetyltrimethylammonium bromide to mediate reverse micelle technology. The morphology of nanorods or nanowires can be changed by coating TbF_3_ on the surface. The morphology of Na (Y1.5Na0.5) F6:Ce:Tb nanomaterials is shown in Figure 2. The obtained Na (Y1.5Na0.5) F6:Ce:Tb nanomaterials can obtain green light emission with the main emission peak at 544 nm under the excitation of ultraviolet light with an excitation wavelength of 256 nm, which is mainly attributed to the ^5^D_4_→^7^F_5_ magnetic dipole transition of Tb^3+^ ions. The sample obtained by the co-doping method of Ce^3+^ and Tb^3+^ causes the energy of Ce^3+^ to transfer to Tb^3+^ due to the energy level matching of Ce^3+^ and Tb^3+^, thereby emitting green light, and the light intensity of the surface coated with TbF_3_ is much stronger than that of the uncoated sample. Gulina et al. (2017) used a gas-liquid interface technique to prepare LaF_3_:Eu^3+^ nanomaterials. The orange-red light with the main emission peaks at 590 and 615 nm was obtained under UV excitation at 394 nm (see Figure 3). The luminescence intensity of the sample increases and then decreases with increasing Eu^3+^ doping concentration, which is a typical behavior of rare earth doped samples: concentration quenching phenomenon. β-NaLaF_4_:Eu^3+^, Gd^3+^ red light nanomaterials were synthesized by the solvothermal method by Xie et al. (Nie et al., 2017) (see Figure 4). The addition of Gd^3+^ improves the red light emission intensity, which they believe is due to the Gd^3+^ entry of β-NaLaF_4_:Eu^3+^ nanocrystals in the lattice gap, which affects the symmetry of the local crystal field. Zeolites have gradually attracted attention because of their microporous nature and their properties in terms of adsorption and ion exchange. In 1948, Barrer et al. (1948) synthesized artificial zeolites for the first time, providing a solid basis for the later diversification of molecular screening species and their widespread use in science and industry (Barrer and Hinds, 1950; Weitkamp, 2000). The phenomenon that the luminescence intensity of the luminescent material decreases as the temperature increases is called temperature quenching. Xiangfu Wang et al. (2015a). found that the luminescence of Er^3+^, Tm^3+^, Ho^3+^, Nd^3+^, Dy^3+^, and Eu^3+^ doped phosphors is very weak at temperatures great than 773 K, due to intense thermal quenching. It is difficult to measure temperature change in the range of more than 500°C through the conventional optical temperature sensing technology. New phosphors with low thermal quenching rates and high luminescence efficiency should be synthesized cheaply. Instead of rare-earth ions, new luminescence centers with high luminescence intensity at high temperature (>773 K) may be excellent candidate activated ions in novel fluorescence sensors. Yu et al. (Wu et al., 2009) of Shanghai Normal University exchanged Eu^3+^ with 13X zeolite using a water bath ion exchange method to prepare red phosphor. The emission wavelength of this red phosphor is 612 nm, and the optimal excitation wavelength is 397 nm. These works lay a foundation for the subsequent application of UV-excited LEDs devices.

**FIGURE 5:**
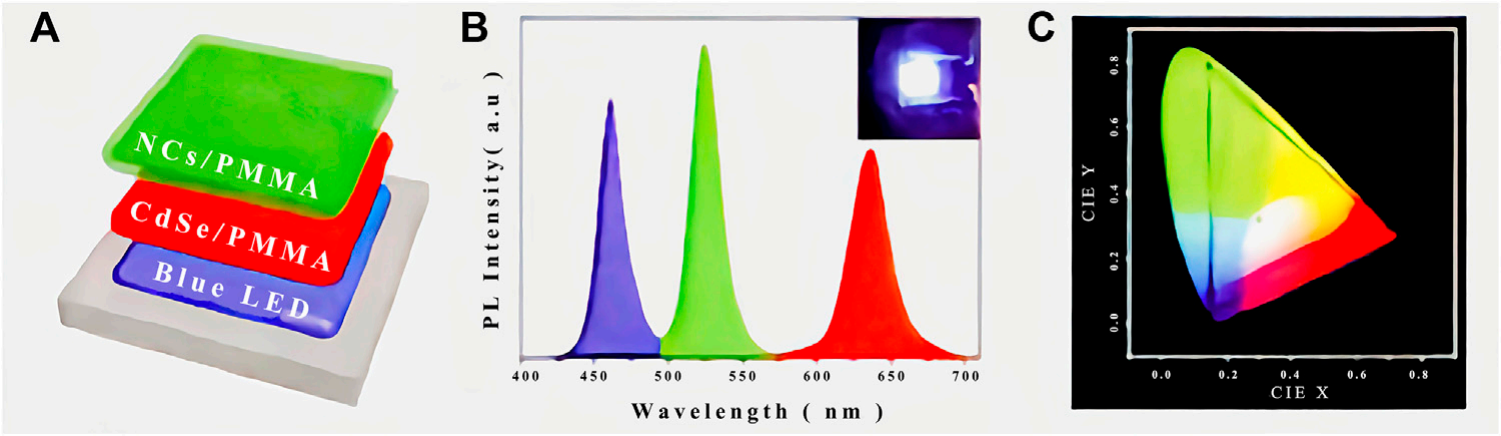
**(A)** Schematic illustration of the configuration, **(B)** PL spectra (Inset shows a photograph of the device) and **(C)** CIE color coordinate of the CsPbBr3/SiO_2_ based WLED (Li et al., 2015).

Micro-nano emitting materials are mainly used in the design and preparation of various micro-nano emitting devices such as micro-nano emitting diodes or micro-nano lasers, which can achieve light-emitting properties not available in macroscopic bulk materials, and also have a wide range of applications in bio-imaging and food safety detection. Light Emitting Diodes (LEDs) are electroluminescent semiconductor devices that are sealed by encapsulants to convert electrical energy into light energy, and they are the focus of research in the fields of materials science, physics and chemistry, and will become the next generation of Solid State Lighting (SSL) devices (Li et al., 2015). Jone’s group (Jang et al., 2008) prepared TOP/TOPO/HAD-coated CdSe red-emitting quantum dots by coating CdSe quantum dots with trioctylphosphine (TOP), trioctylphosphine oxide (TOPO) and cetylamine (HDA). They also prepared Sr3SiO_3_:Ce^3+^, Li+ green light-emitting materials, and then mixed the two light-emitting materials to assemble white LED devices which were excited by blue chips, with a color rendering index higher than 85. They also assembled red LED devices excited by blue light chips based on TOP/TOPO/HAD-coated CdSe red quantum dots. The spectrograms and luminescence photographs are shown in Figure 5. In 2015, Zeng Haibo’s team (Song et al., 2015) reported for the first time the preparation of yellow, green and blue LEDs based on all-inorganic Chalcogenide quantum dots (CsPbX_3_) (X = Cl, Br, and I), proving that all-inorganic chalcogenide quantum dots as a new optoelectronic material has great application prospects in the field of optoelectronics, and the quantum efficiency (EQE) of all-inorganic chalcogenide nano crystal light-emitting diodes rapidly developed from less than 1%–21.3%. In 2019, the team of Gao Feng and his co-workers (Xu et al., 2019) reported the results of molecular passivation for the preparation of high-efficiency chalcogenide light-emitting diodes, which were optimized to achieve an EQE of up to 21.6% for red chalcogenide diodes, the highest external quantum efficiency reported for red chalcogenide light-emitting diodes. In 2020, Edward H. Sargent’s team and his co-workers (Dong et al., 2020) used a surface repair treatment of chalcogenide quantum dots to achieve a bipolar shell consisting of an inner anionic shell and an outer cationic and polar solvent molecular shell. The outer shell is electrostatically adsorbed onto the negatively charged inner shell. This method produces high domain limiting chalcogenide quantum dot solids with improved carrier mobility and reduced defect density, resulting in photoluminescence quantum yields in excess of 90% for blue quantum dot films. By exploiting the improved mobility, highly efficient blue and green light diodes based on CsPbBr_3_ quantum dots were fabricated. After optimisation, the blue chalcogenide light-emitting diodes have an EQE of up to 12.3%. This is the highest exo-quantum efficiency reported for blue chalcogenide light-emitting diodes. On 23 February 2021, Yang Xuyong’s team at Shanghai University and his collaborating team Andrey L. Rogach’s team at City University of Hong Kong (Kong et al., 2021) published in Nature Communications their research on the use of methane sulfonate to smooth energy transfer pathways in quasi-2D chalcogenide films for the preparation of highly efficient LEDs. Reconstruction of quasi-2D chalcogenide structures using strong hydrogen bonding between methane sulfonate (MeS) and BA to increase the energy acceptor to donor ratio and enhance energy transfer in chalcogenide films, resulting in improved luminescence efficiency. The MeS additive also reduces the defect density in RP chalcogenides due to the elimination of unliganded Pb^2+^ by the electron-rich Lewis base MeS and the weakening of the adsorbate blocking effect. As a result, green LEDs prepared using these quasi-2D RP chalcogenide films achieved a current efficiency of 63 cd/A and 20.5% EQE, which is the best performance to date for quasi-2D chalcogenide-based devices and the highest exo-quantum efficiency reported for green chalcogenide light-emitting diodes. In 2021, the team of Ziming Chen and Xuanli Ye (Chen et al., 2021) reported the results of realizing white light-emitting diodes of chalcogenide with an external quantum efficiency of more than 12% through near-field optical coupling. The blue chalcogenide LEDs were optically coupled to red chalcogenide nanocrystals in the near field by means of rationally designed multilayer translucent electrodes to construct high-performance white chalcogenide LEDs with high light extraction efficiency. The red chalcogenide nano-crystal layer allows the extraction of waveguide modes and surface equipartition exciton polarization modes captured in the blue chalcogenide diode and their conversion to red light emission, thereby increasing the light extraction efficiency by 50%. At the same time, the complementary emission spectra of blue photons and down-converted red photons contribute to the formation of white light-emitting diodes. Finally, the device has an external quantum efficiency of over 12% and a brightness of over 2000 cd/m^2^, both of which are the highest in the field of chalcogenide white light emitting diodes.

**FIGURE 6:**
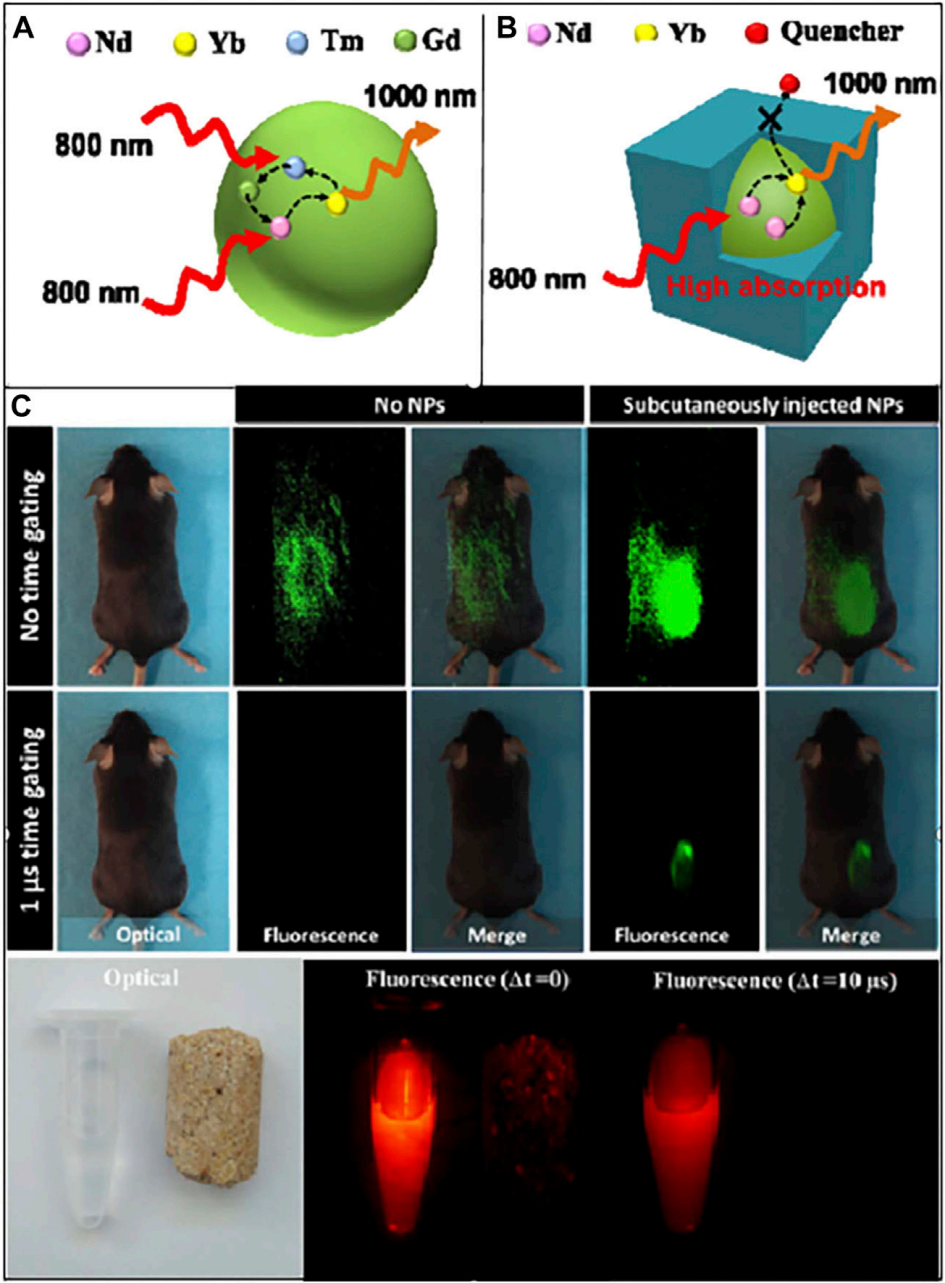
**(A)** Schematic diagram of energy transfer in NaYF4:Yb^3+^, Nb^3+^, and Tm^3+^NPs, **(B)** the core/shell structure of NaYF4:Yb^3+^, Nb^3+^@CaF_2_NPs, **(C)** optical and NIR images of C57BL/6 mice and NaYF4:Yb^3+^, Nb^3+^@CaF_2_ (George et al., 2013).

**FIGURE 7:**
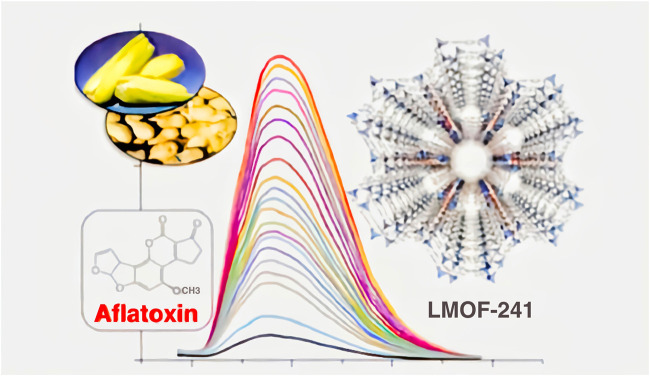
Emission spectra of LMOF-241 with the incremental addition of aflatoxin (Tan et al., 2018).

According to statistics from the US Department of Energy in 2012, LEDs are the most important green and energy-saving light sources in the 21st century. It will save at least 20% of the total US national electricity consumption each year form 2010 to 2030 (George et al., 2013), which shows that LEDs have great potential for future development. For bioimaging, Tan et al. (2018) from Harbin Institute of Technology prepared NaYF_4_:Yb^3+^, Nb^3+^@CaF_2_ core-shell structured luminescent nanoparticles and investigated their luminescence mechanism, and the resulting samples were applied to live imaging in the second infrared window (e.g., Figure 6) with better performance than commercial AgS_2_ nanoparticles. They injected the resulting core-shell structured luminescent material into six rats at the same time and used the laser-pulsed material to emit light and observe intracellular conditions. For food safety detection, Li et al. (Hu et al., 2015) synthesised a highly efficient strong blue light metal organic framework luminescent material (LMOF-241) and used it to detect aflatoxin in food, showing that it was responsive to aflatoxin B1 at one part per billion (Figure 7).

### Micro-Nano Optical Waveguide Materials and Devices

Micro-Nano optical waveguides are the most fundamental units for studying the mechanism of micro-nano photonics and designing micro-nano photonic devices, which have become a hot research topic today and have received great attention in related fields (van Eijkelenborg et al., 2003; Noginov et al., 2009; Khatua et al., 2011; Knight et al., 2011; Sivis et al., 2013). Common micro-nano optical waveguides mainly include micro-nano optical fibers (Miura et al., 1997; Brambilla et al., 2009; Brambilla, 2010), silicon-based planar waveguides (Jalali et al., 1998; Lee et al., 2001; Celler and Cristoloveanu, 2003; Xia et al., 2007), semiconductor nanowires (Sirbuly et al., 2005), and metal surface equipartition excitonic waveguides (Ozbay, 2006). Micro-Nano fibers have sub-wavelength sized fibers diameter and their optical properties present a great difference from conventional fibers. This sub-wavelength scale micro-nano fiber is not only easy to prepare, but also has a series of advantages, such as extremely low optical transmission loss (Brambilla et al., 2004; Brambilla et al., 2006), large waveguide dispersion (Holmes et al., 2000; Mingo, 2003), strong optical field confinement (Polynkin et al., 2005; Villatoro and Monzón-Hernández, 2005), strong abrupt field propagation properties (Brambilla et al., 2007), and easy integration with existing systems (Liu et al., 2011), so it has a very broad application prospect and good potential in optical communication, optical sensing, optical filtering and super continuous spectrum generation.

Micro-Nano fibers can be divided into solid-core fibers and hollow-core fibers according to their structure. Solid-core optical fibers are mainly glass optical fibers and crystal optical fibers. In the 1980s, British scientists such as Boys (1887), in order to analyze the mechanical properties of glass filaments, heated the ore to a molten state and drew it into 100 um filaments, which was the earliest preparation of optical fibers in the world. In 1959, American scientist N.S. Kapany prepared glass filaments with an average diameter of 2.5 nm in the laboratory, which was the first report of the use of micro-nano optical fibers for the transmission of light and images. Constrained by factors such as fiber transmission loss, this glass fiber was difficult to get practical (Kapany, 1959), and for many years after this, no substantial progress was made in the field of micro-nano fiber research. It wasn’t until1966 that Dr. Gao pointed out that optical fibers could be used in optical communications when their transmission loss was reduced by 20 dB/km, and that the theoretical value of attenuation loss could be as low as a limit of 0.1 dB/km, and that it was extremely possible to pull ultra-low loss fibers for optical communications by improving manufacturing equipment and processes (Kao and Hockham, 1996). In 1970, Corning produced the first practical optical fiber with a loss of only 20 dB/km, which allowed people to see the dawn of using optical fibers for communication (Kapron et al., 1970). After the 1970s, scientists began to experiment with the use of flame or laser heating to stretch high-quality glass fibers, resulting in micro-nano waveguides with smooth surfaces and homogeneous structures (Burns et al., 1986; Love and Henry, 1986; Black et al., 1988). In 2003, Professor Tong Limin of Zhejiang University proposed the famous “two-step stretching method,” using sapphire rods as an aid to produce glass fibers with uniform and uniform diameters down to 50 nm, with a loss of only 0.1 dB/mm in this micro-nano fiber (Tong et al., 2003). By improving the experimental setup, Tong et al. (2005) proposed a self-modulated two-step method and succeeded in obtaining a micro-nano fiber with an even lower transmission loss of only 20 nm. In order to solve the disadvantages of the self-modulated two-step process, such as the difficulty of reproducibility, high manual dependence and difficulty in controlling the accuracy, Brambilla et al (2004) used a stepper motor to control the moving speed and used oxygen and isobutane flames to heat the cone region of the fiber to draw ultra-low loss nanowires with a diameter of 320 nm and a loss of about 0.01 dB/mm. In 2004, Leon-Saval et al. (2004) succeeded in drawing a 950 nm diameter micro-nano fiber with a loss of only 0.0014 dB/mm at a wavelength of 1,550 nm. After research, Brambilla et al. (2006) again reduced the diameter of the fiber to 60 nm, reducing the loss to 0.001 dB/mm. In 2006, Tong et al. (2005) successfully prepared glass fibers doped with rare-earth ion disks, silicates, tellurates and fluorides by focal melting, making the fabrication of micro-nano optical fibers no longer limited by raw materials and achieving a leap from passive to active micro-nano photonic devices. First proposed in the 1850s (Christopher, 1998), hollow-core fibers are a new type of waveguide structure with an air core. The main tool for fabricating hollow-core fibers is two different inner diameters of capillaries, which can be well used for making sub-wavelength diameter fibers due to their small inner diameter, which is almost the same as the inner and outer diameters of ordinary fibers. The first successful hollow-core energy transfer fibers were manufactured in the 1960s, mainly from vulcanized glass and metals, but the hollow-core fibers produced at that time had obvious defects in both structure and material selection, and had significant transmission losses that prevented their use in practice (Kubo and Hashishin, 1987; Miyagi and Karasawa, 1990). Mareatillli and Sehmeltzer were the first to develop a systematic theory and analysis of the transmission of hollow-core fibers, summarizing the factors affecting the transmission loss of light in hollow-core fibers and forming the original theory of hollow-core waveguide transmission, the famous M-S theory. In the late seventies and early eighties, according to the classical microwave waveguide structure theory system, Professor Marhic from Northwestern University in the United States conducted a study on the bending loss and mode coupling of optical fibers. Immediately afterwards, Professor Mitagi from Tohoku University in Japan further refined the M-S theory and improved the Miyagi formula with even greater precision. The concept of infrared hollow-core fibers, first introduced by Hidaka et al. (1981) in 1981, has sparked a wave of interest among scientists in the investigation of hollow-core fibers in high-power laser transmission applications. A group led by J.A. Harrington, at Rutgers University, United States, developed a glass hollow-core fiber that could reach 6 m in length (Nubling and Harrington, 1996; Nubling and Harrington, 1997). In 2002, Yushan Yan’s research group at the University of California used natural spider silk as a fabrication tool and a specific process to achieve a hollow fiber structure with a hollow core diameter of only 2 nm in silicon oxide micro-nano hollow core fiber (Wang et al., 2002). In 2015, Habib et al. (2015) from the Technical University of Denmark reduced the loss at mid-infrared 2.85 μm wavelength to 0.002 dB/km through a rational design. In 2016, Habib et al. (2016) designed an elliptical structure of hollow-core anti-resonant micro structured fibers, which can have an extinction ratio greater than 1,000 in broadband 1.0–1.65 μm range, while the leakage loss of the fundamental mode is less than 15 dB/km in the same range. In 2016, Professor Fan Zhongwei’s team from the Institute of Optoelectronics, the Chinese Academy of Sciences (Liu et al., 2016) theoretically demonstrated that circular double-core hollow-core antiresonant micro structured fibers have low limiting loss and elliptical double-core hollow-core antiresonant micro structured fibers have a wide transmission band. In 2017, Habib et al. (2017) designed a low-loss hollow-core antiresonant micro structured fiber with a loss as low as 0.0015 dB/km at wavelength 1.06 μm and 0.006 dB/km when the bending radius was 5 cm, while achieving a high extinction ratio in the 1–1.1 μm wavelength range with a 1,500 extinction ratio of. In 2017, Hayes et al. (2017) from the University of Southampton achieved short-range data transmission with a bandwidth greater than 1,000 nm and a low loss of 25 dB/km at wavelengths 1,065, 1,565, and 1963 nm using a hollow-core anti-resonant micro structured fiber. In 2018, Adaum et al. (2018) from the Technical University of Denmark used argon gas-filled hollow-core anti-resonant micro structured fibers to achieve 200–4,000 nm multi-octave super continuum spectrum through mid-infrared ultrashort pulse pumping. The current hollow-core anti resonant micro structured deflection-preserving fibers are mainly designed theoretically. In 2018, Tu et al. (2018) of Hokkaido University, Japan theoretically proposed two types of As_2_S_3_ glass hollow-core anti resonant micro structured fibers for high-power CO_2_ laser transmission with a loss of 2.6 dB/km at wavelength 10.6 μm, achieving both low loss of less than 1 dB/km and high birefringence of greater than 1× $10^{-4}$ in the broadband range. Wei et al. (2018) designed a polarized low-loss negative curvature hollow-core fiber with a loss of 0.020 dB/m and a birefringence of $\;10^{-4}$ and an extinction ratio of 850. The team led by Professor Shuqin Lou from Beijing Jiaotong University (Yan et al., 2018) designed a single-polarization single-mode double-ring hollow-core anti-resonant micro-structure fiber, which can achieve single polarization in two wavelength bands of 1,545–1,553 nm and 1,591–1,596 nm. At the wavelength of 1,550 nm, the fundamental mode loss of the *x*-axis is only 0.04 dB/m, the extinction ratio is 17,662, and the minimum extinction ratio of the high-order mode is 393.

Compared to solid core fibers, hollow core fibers exhibit superior performance and are mainly used for energy transmission. It has a longer service life, greater transmission power, less loss at the input end, and more stable output optical field characteristics. It can be well used to transmit high-power optical radiation fields, which will bring new opportunities for future optical communication systems, medical equipment, military, sensing and even military fields.

### Micro-Nano Photoelectric Detection Materials and Devices

Photodetectors are like the “eyes” of mankind and play a very important role in the development of human civilization. A photodetector is a photoelectric device that receives light signals from a target or from its own radiation and, through transformation, processing and control, obtains the required information. Its basic function is to convert the received light signal into an electrical signal and achieve some application purposes, such as photoelectric imaging, weather observation, night vision, remote sensing, etc. By the material theory limit of materials and other factors, the traditional detection materials cannot meet the current stage of photodetector device development needs. Therefore, continuously deepening and optimizing the existing material system research, expanding the application direction, while continuing to carry out research and development of new materials, looking for better performance of the detection materials, are the inevitable requirements of the development of photodetector technology. With the rapid development of nanoscience and materials science, hundreds or thousands of new nanomaterials have emerged in the past decade, such as quantum dot (Hwang et al., 2005; Krishna, 2005; Kufer et al., 2015), nanowire (Liu et al., 2010; Zhang et al., 2010; Miao et al., 2014a; Miao et al., 2014b), two-dimensional material (Li et al., 2008; Liu et al., 2012; Furchi et al., 2014; Sun and Chang, 2014; Zhang et al., 2014), nanosheets (Amini et al., 2012; Tian et al., 2017), and dye molecule materials etc. These new nanomaterials have different characteristics from conventional thin film materials, such as nanoscale geometry and large specific surface area in a certain dimension, making them exhibit novel physical and chemical properties and excellent optoelectronic performance. In recent years, two-dimensional van der Waals layered materials have become a strong contender for photo detectable materials for artificial micro-nano structures due to their novel physicochemical properties and excellent optoelectronic performance. Two-dimensional materials are a new class of materials with similar atomic arrangements, scales, and bond strengths in two dimensions, but significantly stronger than in the third dimension. When nanomaterials and device structures are comparable to Fermi wavelengths in one spatial dimension, electrons moving in restricted directions are scattered by boundaries and cannot be seen as classical particles moving in an external field, and the density of electron energy levels near the Fermi plane changes from quasi-continuous to discrete quantized energy levels. The quantum size effect in this low-dimensional material system makes the electron density of states exhibit significantly different low-dimensional characteristics from those of conventional bulk materials, and the physical behavior of carrier transport and optical leap has quantum confinement, resulting in many novel physical properties and effects that are highly promising for new electron transport devices and optoelectronic devices.

**FIGURE 8:**
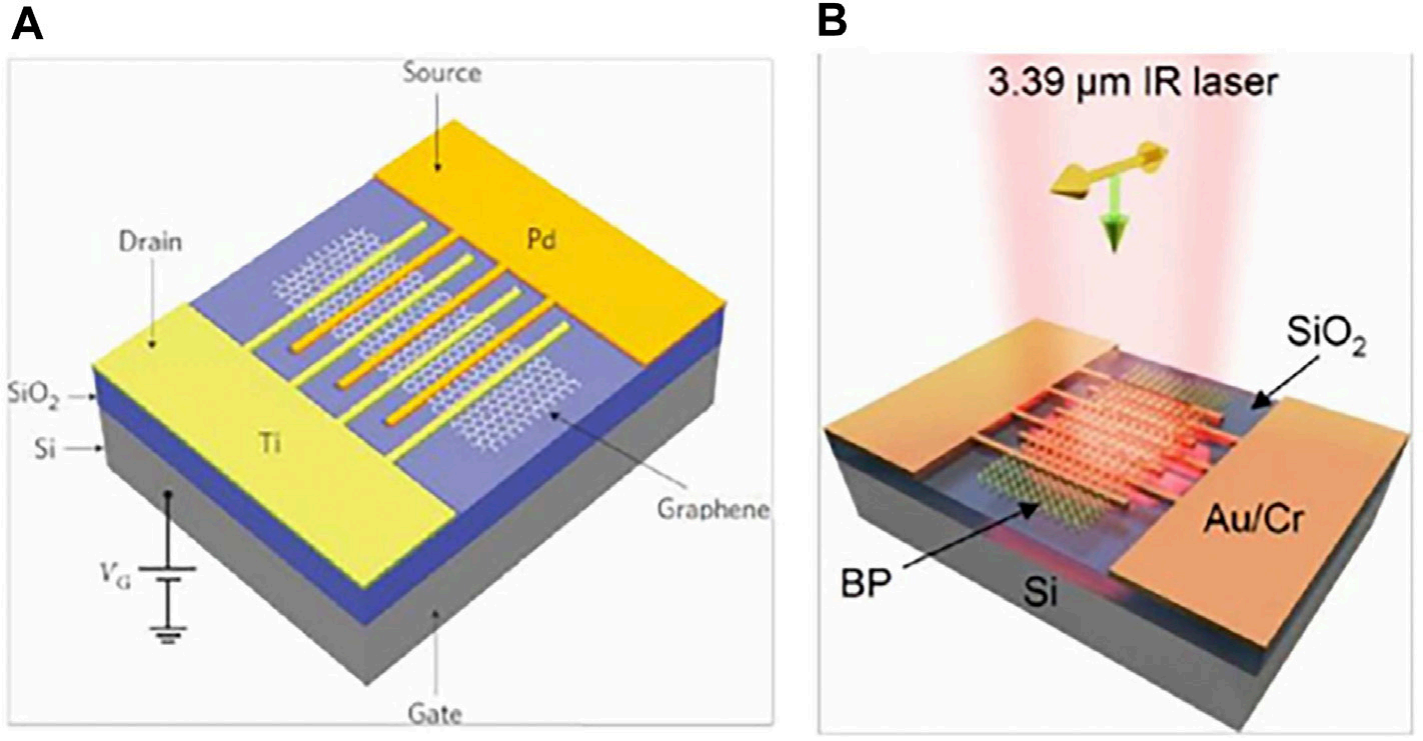
**(A)** MGM photodector with heterogeneous metal interdigital electrode (Mueller et al., 2010); **(B)** BP mid infrared detector integrated interdigital electrode (Guo et al., 2016). (Neto et al., 2007)

**FIGURE 9:**
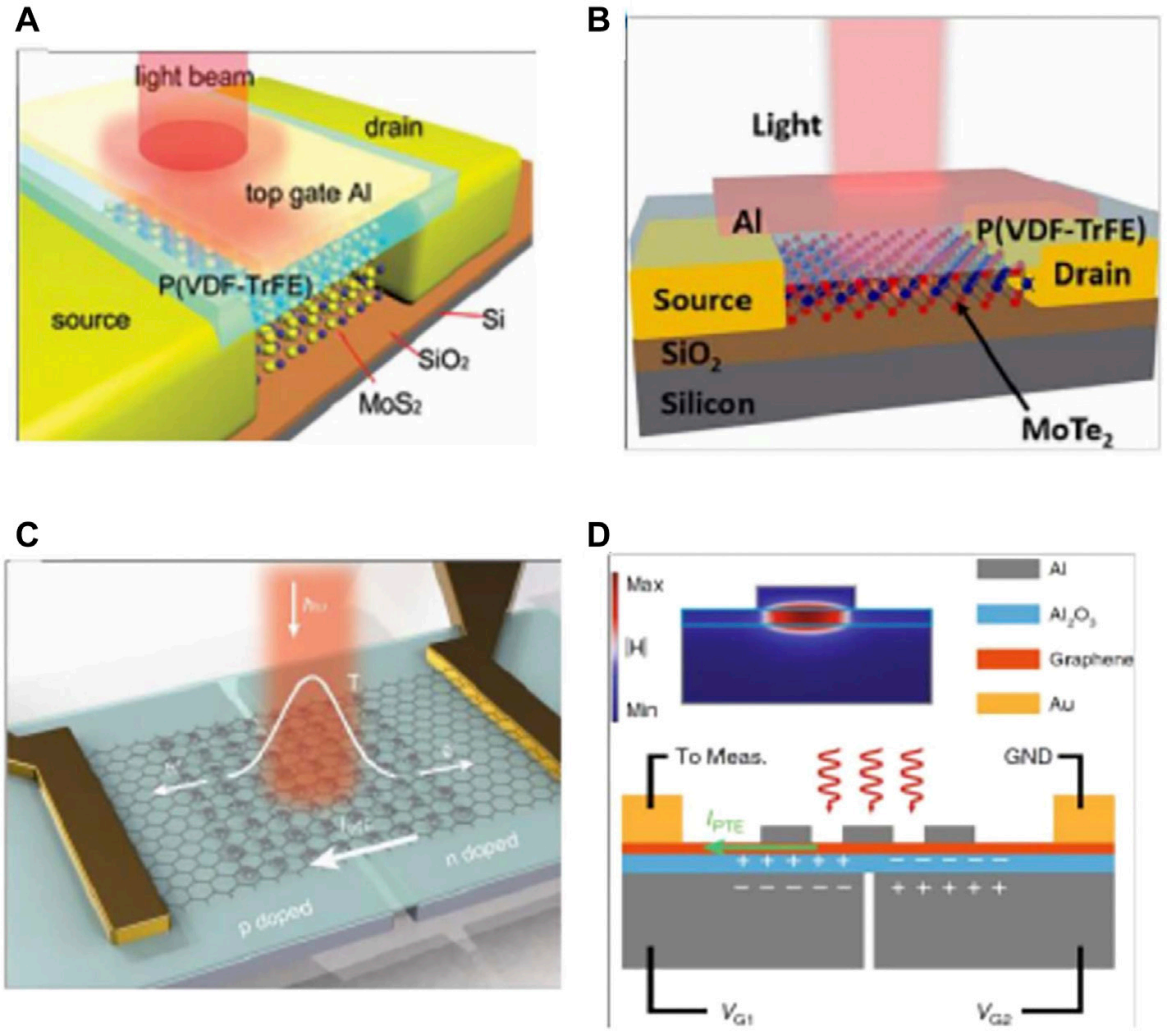
**(A)** Photodetector based on ferroelectric polymer and MoS_2_ (Wang et al., 2015b). **(B)** Photodetector based on ferroelectric polymer and MoTe_2_ (Huang et al., 2016b). **(C)** Plasmonic microcavity integrated graphene photodetector (Wang et al., 2020). **(D)** Cross-sectional schematic of the graphene photodetector with split gates and a nano disk (Wang et al., 2020).

In 2010, Muller et al. (2010) prepared an MGM photodetector that responds to NIR using heterogeneous metals (titanium and palladium) fork-finger electrodes, as shown in Figure 8A. The heterogeneous metal configuration in the fork-finger electrode increases the junction area while breaking the mirror-image energy band bending of the original two adjacent metal/graphene contact surfaces to achieve efficient collection of optoelectronic signals. Under 1.55 μm NIR laser irradiation, the superimposed electron-hole pairs within each junction zone of the device are directionally separated by means of an applied source-drain bias Vds, contributing to the overall current, with a final photo response of 6.1 mA/W. In 2016, Guo et al. (2016) prepared a mid-infrared photo detector using forked-finger electrodes on a 12 nm thick two-dimensional layered BP, as shown in Figure 8B. The staggered short channel of the device is formed by the forked finger electrode and the BP accelerates the carrier’s movement under the action of the applied electric field, reducing the crossing time of the photogenerated carriers and significantly enhancing the photoelectric gain. Due to the presence of a forbidden band in the BP, dark currents can be suppressed to a certain extent and weak light detection is achieved, with the final device achieving a photoresponse of 82 A/W in the mid-infrared band at 3.39 m. In 2016, Wang et al. (2015a) of Shanghai Institute of Technology combined ferroelectric polymer P (VDF-TrFE) with MoS_2_ to prepare a high performance FET photodetector, and the device schematic is shown in Figure 9A, with the ferroelectric polymer acting as a floating gate to modulate MoS_2_. Since the ferroelectric polymer can generate an ultra-high local electrostatic field on the semiconductor material through polarization, this property enabled the first extension of the light detection capability of the MoS_2_ from the visible to the near-infrared (0.85–1.55 μm). In 2016, Huang et al. (2016b) prepared a MoTe_2_ transistor detector with a ferroelectric polymer as the top gate, as shown in Figure 9B, where the residual polarization of the ferroelectric body can be used to deplete MoTe_2_ without gate voltage Vg, significantly improving the device’s performance. Under the effect of ferroelectric polymer polarization, MoTe_2_-based FET devices can achieve broad spectrum detection (600–1,650 nm) from the visible to the near infrared, with an optical responsivity of 16.4 mA/W and a detection rate of 1.94×10^8^ Jones at a wavelength of 1,060 nm. In 2020, Wang et al. (2020) elaborated a graphene-bound plasmonic microcavity photodetector. As shown in Figure 9C, the bottom metal is a double-gate structure, and graphene p-n junctions can be artificially constructed by applying different gate pressures. In this composite structure, the bottom metal Al, the middle dielectric layer Al_2_O_3_, the monolayer graphene and the top gold nano-disc together form a plasmonic microcavity (Figure 9D), which excites the graphene plasmonic resonance and heats up the carriers by modulating the local state of the optical field. The result is a composite detector with a photo thermal effect (PTE) of 51.99 A/W (incident wavelength 638 nm). With this photo-electric coupling mode, the photocurrent response is enhanced by a factor of 25 one compared to a normal device. In 2018, Wang et al. (2018) proposed an ultra-thin class II superlattice detector structure. The thickness of the detector IR absorbing material is 1/50th of the wavelength, and by introducing heavily doped InAs and a surface nano-antenna structure, the incident light at the resonant wavelength is localised in the ultra-thin absorber layer, achieving nearly 50% of the detector absorption. Feng et al. (2019) from Fudan University proposed an improved all-Si thermo electronic photodetector structure using electron-beam lithography self-alignment to integrate a specially designed super-surface as an antenna into an array of silicon nanowires on an insulator. The responsivity and detectivity of the detector at 1.15 μm and 480 nm bandwidths are as high as 94.5 mA/W and 4.38 × 10^11^ cm Hz^1/2^/W, respectively. In 2020, Nordin et al. (2020) proposed and fabricated a class II superlattice LWIR detector structure with an integrated heavily doped semiconductor, exploiting the metal plasma properties of the heavily doped semiconductor (1 × 10^19^ cm^−3^ doping concentration) to form a resonant cavity to enhance the absorption of long-wave infrared radiation at resonance. The structure is designed to enhance the absorption of long-wave infrared light at resonance. Two detectors were designed with a target absorption wavelength of 8 and 10 μm, with a total absorber layer thickness of only 1.42 and 1.80 μm, respectively. 45 and 27% peak external quantum efficiencies were achieved for the two devices, respectively. In 2021, Kamboj et al. (2021) in the D. Wasserman’s group also reported a fully epitaxially guided mode resonant mid-wave IR type II superlattice nBn photodetector consisting of a high refractive index absorber/waveguide layer, a low refractive index semiconductor absorber layer and a heavily doped reflector layer. The experimental results show that the absorption is strongly enhanced at the wavelengths coupled to the guided mode resonance. For TE polarised light with an absorber layer thickness of only 250 nm, the detector achieves an external quantum efficiency of more than 50% and a detection rate D* of 4×10^10^ cm Hz^1/2^ W^−1^ at a high operating temperature (T = 200 K).

### Micro-Nano Optical Structures and Devices

Micro-nano optical structure technology refers to the realization of new optical functional devices by introducing micro-nano optical structures into materials. Photonic crystal is a regular three-dimensional microstructure, which refers to an ordered structure material formed by two or more dielectric materials with different dielectric constants (refractive indices) arranged in a certain periodic order in space. The scale of repeating building blocks is on the order of wavelengths of light. The so-called “crystal” is not a crystal formed by the periodic distribution of molecules, atoms or ions in the usual sense, but refers to the “periodicity” of the microstructure. The spatial period scale of photonic crystals is comparable to the wavelength of visible light, and the Bragg diffraction caused by the structural periodicity can generate a “photonic band gap,” also called a photonic band gap, within a certain frequency range. Electromagnetic waves with frequencies in the photonic energy band can propagate almost losslessly in photonic crystals, but electromagnetic waves in the photonic forbidden band cannot propagate in photonic crystals. Therefore, the main optical feature of photonic crystal materials is the photonic band gap. By rationally designing the material composition, effective refractive index, lattice parameters, etc. of the photonic crystal, photonic crystals with photonic band gaps in specific wavelength bands can be artificially prepared. Compared with electrons, light can carry more information, have a wider band gap, and reduce energy loss. As a new type of optical material, the existence of photonic band gaps endows photonic crystals with very important application value, which can be used to fabricate high-performance optical devices that were impossible to fabricate before, such as straight (acute) angle optical waveguides, photonic crystal fibers, low-closure optical devices Value laser oscillators, integrated optical circuits, thermoelectric systems and biochemical sensors, etc.

Photonic crystals are periodic arrangements of different dielectrics in one, two or three dimensions, and Bragg diffraction occurs when light propagates in this periodic medium. Referring to the method of crystal research, the space period of photonic crystal is divided into a family of parallel and equally spaced plane lattices (hkl), then the Bragg equation can be expressed as: mλ=2ndhklsinθ . Among them, m is the Bragg diffraction order and the diffraction wavelength, n is the average refractive index of the photonic crystal material, d_hkl_ is the coordinate (hkl) interplanar spacing, and θ is the Bragg diffraction angle. The properties of Bragg diffraction of photonic crystals are similar to the forbidden band theory of photonic crystals: Bragg diffraction is the reason for the optical forbidden band of photonic crystals. In photonic crystals, light waves in the forbidden band cannot propagate in any direction due to Bragg diffraction in all directions. If the periodicity of the dielectric arrangement at a certain position in the photonic crystal is broken, the light waves can be emitted from this defect, which is the principle for the application of photonic crystals for signal transmission.

In 1987, John (1987) and Yablonovitch (1987b) first proposed the theory of photonic crystals. According to the periodic structure characteristics of different directions in space, photonic crystals are divided into one-dimensional, two-dimensional, and three-dimensional photonic crystals. One-dimensional photonic crystals are the simplest photonic crystals. It only requires the distribution of periodic components of the same refractive index material in one direction, and the other two directions have a periodic qualitative multilayer structure, and the photonic band gap can only exist in one direction. In 2013, Yan et al. (2013) studied the influence of micro-nano fiber diameter and incident wavelength on electric field and energy distribution through theoretical calculation and simulation. The results show that as the incident wavelength decreases, the electric field mode and the time-averaged energy density in the fiber increase at the same core diameter. At the same incident wavelength, with the increase in the diameter of the fiber, the time average value of the electric field mode and the energy density also increases, and when the diameter increases to be equivalent to the incident wavelength, the maximum value is reached, and then as the diameter continues to increase, the average value of the electric field mode and energy density of the fiber is reduced again. In addition, when the diameter of the micro-nano fiber is constant, the energy confined in the core is inversely proportional to the incident wavelength. The shorter the incident wavelength, the more energy is confined in the core. When the incident wavelength is constant, the energy density in the fiber core is proportional to the fiber diameter, that is, as the fiber diameter increases, the energy confined in the fiber core gradually increases. In 2019, Xiang (2019) conducted research on III-V nanowire lasers based on photonic crystal microcavities, designed an In0.53Ga0.47As nanowire laser based on photonic crystal microcavities, and compared mode field distribution and threshold gain of microcavity nanowire lasers. The results show that in the absence of a photonic crystal microcavity, as the diameter of the nanowire decreases, the confinement ability of the light field is weakened and the threshold gain is increased, and it is impossible to excite when the radius is less than 140 nm. Due to the strong mode confinement effect of the photonic crystal microcavity, the lasing cut-off radius of the In0.53Ga0.47As nanowire laser based on the photonic crystal microcavity is reduced to 70 nm. A two-dimensional optical crystal refers to a material whose dielectric constant is distributed periodically in one plane, while its structure is uniform in the third dimension, and there are photonic band gaps in two directions. A three-dimensional photonic crystal refers to a dielectric material whose dielectric constant changes periodically in three directions. If the structural parameters are selected properly, it is possible to have an omnidirectional photonic band gap, and after entering the photonic crystal, some specific frequencies of light are prohibited from propagating in all directions. Although this structure has many advantages, it is not easy to manufacture, especially it is difficult to achieve below the order of millimeters.

**FIGURE 10:**
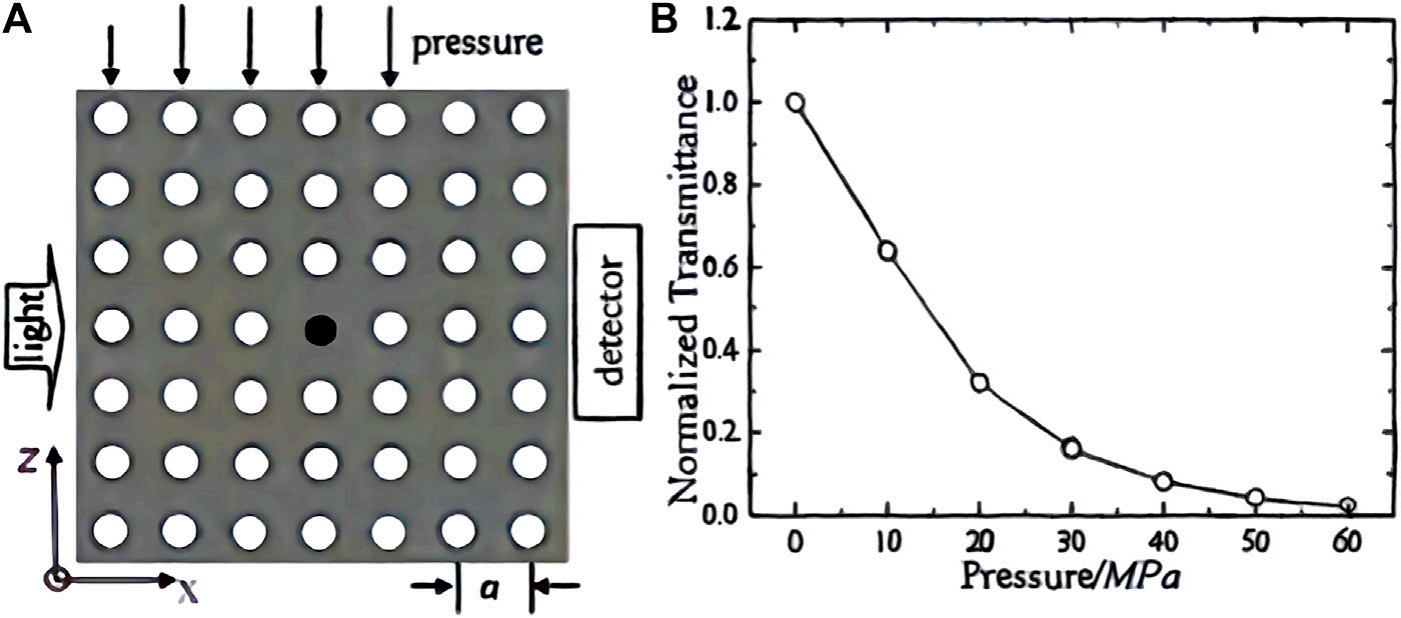
**(A)** 2D-PC thin film point defect structure; **(B)** Relationship between transmittance and pressure (Zhao, 2015).

**FIGURE 11:**
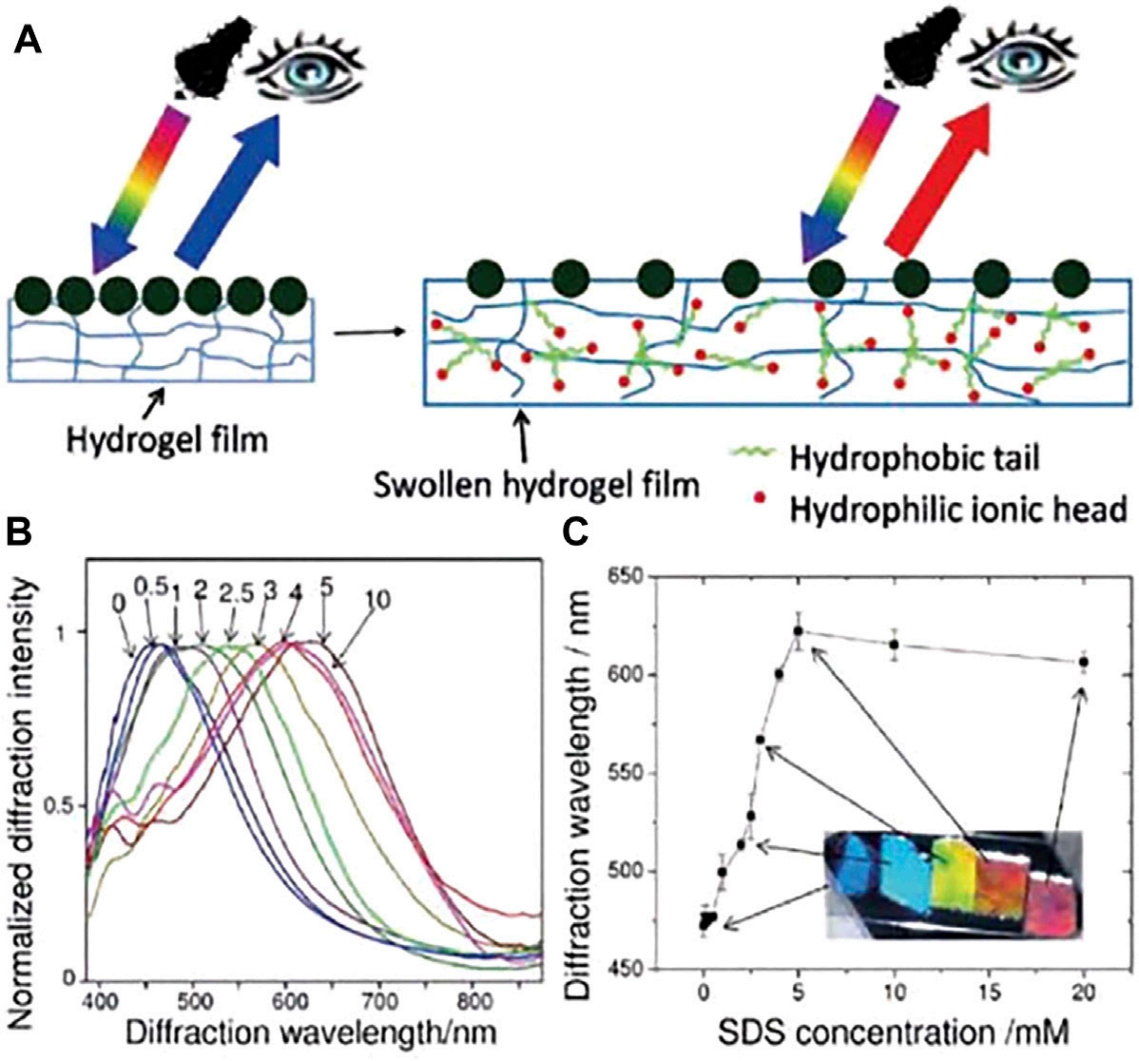
**(A)** Schematic of a 2D-PC sensor formed by polymerizing a PNIPAAm hydrogel onto a single-layer microsphere array. The PNIPAAm hydrogel swells after binding to the surfactant molecule, and the ball spacing increases, resulting in diffraction red-shift; **(B)** The normalized diffraction spectrum of the 2D-PC PNIPAAm sensor in aqueous solution of sodium dodecylsulfonate (SDS) at different concentrations; **(C)** The diffraction wavelength to the SDS concentration response and corresponding color change (Zhang et al., 2012).

**FIGURE 12:**
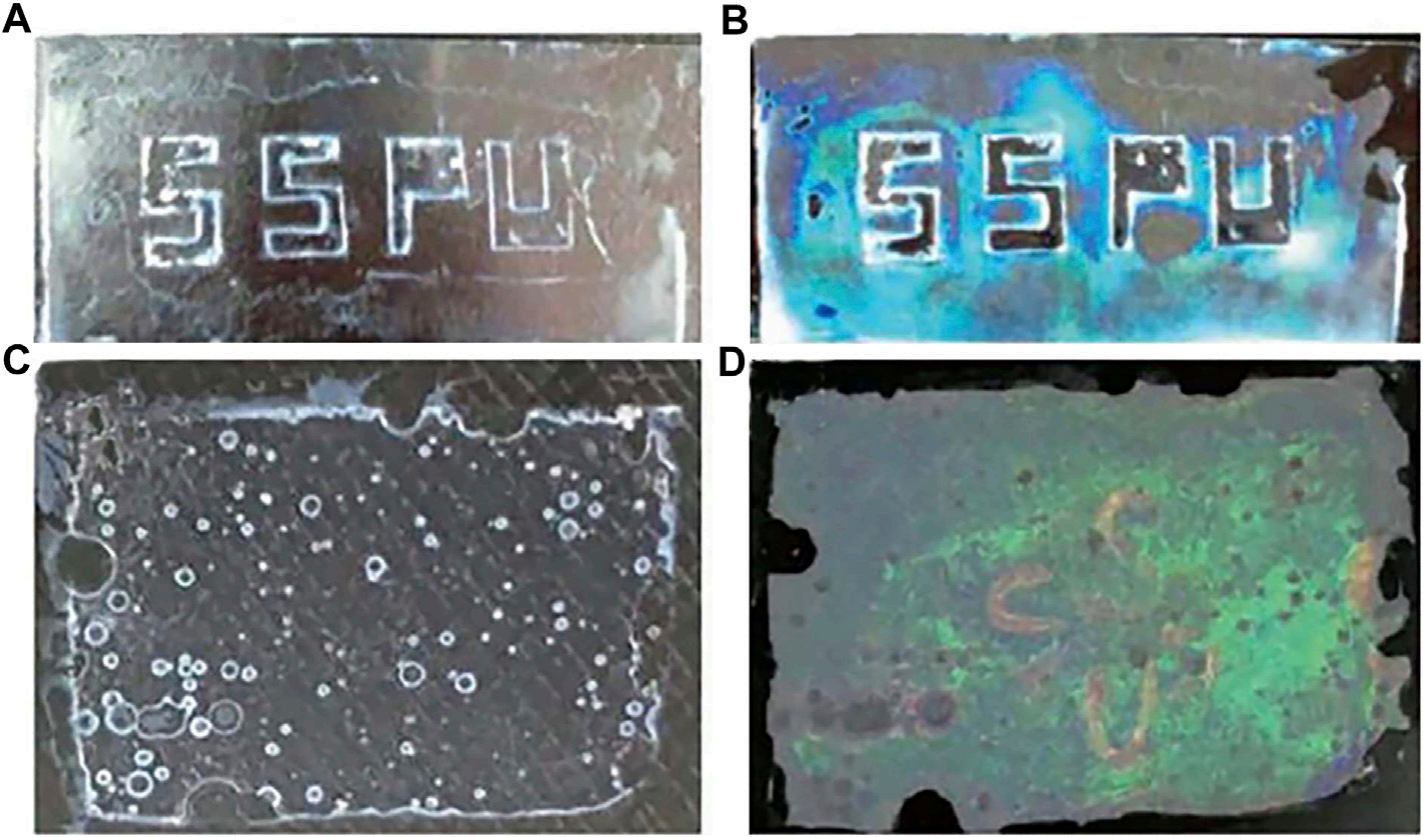
2D-PC material with invisible markings: **(A)** colorless and transparent when dehydrated; **(B)** PC film with blue and green when rehydrated; **(C)** colorless and transparent when dehydrated; **(D)** The PC film turned green while the printed part showed red (Chen et al., 2017b).

Currently, three-dimensional photonic crystals are favored by researchers because of their ability to produce omnidirectional photonic forbidden bands, so their preparation and applications are also more mature, and their applications cover communication propagation, intelligent sensing and other fields. For example, Chen et al. (2010) immobilised photonic crystal arrays in a polyvinyl alcohol (PVA) hydrogel matrix by physical cross-linking to effectively modulate the diffraction wavelength in the visible region, and based on this, developed a series of sensing materials that rapidly respond to a variety of environmental stimuli such as solvents, pH, glucose, etc. The resulting color changes can be easily discerned by the naked eye (Chen et al., 2015; Chen et al., 2017a; Ruan et al., 2017); Xiao et al. (2017) developed a smart photonic crystal hydrogel material that can be used for the real-time monitoring and removal of uranium ions (UO_2_^2+^). The introduction of defects in photonic crystals can form special materials for applications in waveguides (introduction of line defects), lasers (introduction of point defects), etc. However, the precise introduction of defects in three-dimensional photonic crystals is difficult with the current state of the art, and the preparation of three-dimensional photonic crystals is more complex. As semiconductor planar technology has been developed more maturely, two-dimensional photonic crystals have the advantage of being easier to prepare and easier to introduce defects than one- and three-dimensional photonic crystals, and therefore, two-dimensional photonic crystals are gradually entering the vision of researchers. Using the characteristics of 2D photonic crystal point defects, Zhao (2015) modelled a 2D photonic crystal point defect thick plate structure, which can change the transmission spectrum of the thick plate by changing the pressure, as shown in Figure 10, and theoretically demonstrated the feasibility of 2D photonic crystal as a pressure sensing material. Unlike traditional photoelectric sensors, photonic crystal sensors do not require complex circuitry and large structural equipment, and are simple in structure and can discern changes in physical quantities by naked-eye observation. Zlatanovic et al. (2009) used the principle of photonic crystal microcavity structure to prepare a photonic crystal microcavity sensor for quantitative detection of trace proteins. The sensor operates with energy concentrated in the photonic crystal microcavity structure, and changes in the microcavity structure can have a significant effect on the output spectrum. When the carboxyl group of the functionalised group in the microcavity is combined with the biotin bovine serum albumin molecule (b-BSA), the resonance wavelength in the microcavity changes, and when the concentration of b-BSA changes, the concentration can be detected by measuring the standard curve of the change in the wavelength position of the resonance peak. Zhang et al. (2012) developed a two-dimensional photonic crystal sensing material for visual detection of anionic and cationic surfactant concentrations in water, which is a hydrogel film based on a two-dimensional microsphere array of poly (N-isopropylacrylamide) (PNIPAAm) that can detect surfactant concentrations down to 0.1 mM in aqueous solutions (Figure 11). Men et al. (2016) attached Au nanosphere arrays to poly (acrylic acid) (PAA) hydrogel membranes to prepare stand-alone two-dimensional Au nanosphere array/hydrogel composite sensing membranes. The Au nanosphere array/hydrogel composite membranes showed visual diffraction colors and enhanced diffraction intensity due to the periodic structure and the large scattering cross-section of Au nanospheres, and the proposed strategy can be extended to various functional hydrogel membranes to develop different visualization sensors. Similarly, a range of optical devices can be designed and developed using the material’s response to environmental stimuli to produce volume changes that alter the crystal plane spacing of 2D photonic crystals. Chen et al. (2017b) prepared a two-dimensional photonic crystal film material for invisible markers, as shown in Figure 12, which can be used for controlled color development or anti-counterfeit printing by not only going from transparent to visible, but also revealing pre-printed patterns when wetted with water. Dalstein et al. (2016) developed a soft lithography method for fabricating two-dimensional photonic structures of submicron metal-organic backbones (MOF). ZIF-8 (zinc) is a suitable MOF material due to its good chemical stability and vapour-selective adsorption properties. Combining these photonic MOF heterostructures with smartphone technology allows for the development of very low-cost sensing and monitoring platforms. The method can detect changes in the diffraction efficiency of photonic MOF patterns due to changes in the MOF refractive index by a charge-coupled device (CCD) camera, which is integrated into the smartphone and does not require complex optical instrumentation to transmit the data. In 2016, Jiaming Shi et al. of the School of Electronic Countermeasures proposed a one-dimensional Si-doped film of ZnSe/Te photonic crystal (Miao et al., 2016) to achieve an infrared camouflaged selective radiator for laser and double detectable windows by doping Si films. In 2017, his team introduced a Si film inside a Ge/ZnS photonic crystal (Zhang et al., 2017) to propose a one-dimensional doped structured photonic crystal to achieve IR-laser-radar compatible stealth. In 2019, his team optimized the visible-IR compatible camouflage design for the ZnSe/Ge system (Zhang et al., 2019), and the thickness of the proposed dual-band IR camouflage selective radiator was further optimized to 7.8 μm. In 2021, Gong Rongzhou’s team at Huazhong University of Science and Technology prepared a one-dimensional photonic crystal and covered it with a thin film of phase change material GST, and such photonic crystal has an adaptive effect of infrared camouflage (Su et al., 2021).

Two-dimensional photonic crystal sensors have the advantage of compact structure compared to traditional sensors, which is more conducive to the miniaturization and portability of sensors. Two-dimensional photonic crystal sensors will become a research hotspot in the field of sensor technology in the coming period, and will then have a wide range of applications in fields such as the Internet of Things and biomedicine, especially in the area of flexible wearable sensing and display devices. This also places higher demands on the reusability, sensitivity and fast response to environmental parameters of photonic crystal materials.

## Conclusion

Optical devices with feature sizes up to the micro-nano scale give rise to many new phenomena that are not available under macroscopic conditions. The optical properties of micro-nano structures allow the design of new optical devices and systems, but the technology for the preparation of micro-nano optical materials and structures has become a technical bottleneck in the development of micro-nano optics. Research on optical micro-nano structures has been carried out for more than 20 years, many problems have been overcome, and many preparation methods and applications have been developed. However, the preparation of optical micro-nano structures has been costly and time consuming, and for optical structures responding to visible wavelengths or even shorter wavelengths, microfabrication means are even more limited and expensive. These reasons inevitably prevent the excellent properties of optical micro-nano structures from being used on a large scale in industry. In addition, the size or structural parameters of almost all current optical micro-nano structures cannot be changed once they are set during the preparation process and therefore have a fixed optical effect. In practical production applications, there is a desire to obtain devices that can be self-executing and adjusted in real time to achieve more functions. The question of how to achieve a large degree of tuning output in the history of optical micro-nano structures is still an issue worth investigating. Finally, the properties of optical micro-nano structures should not only be limited to the control of light transport processes, but also how to effectively apply their advantages to industries related to people’s livelihood, such as LED lighting and solar energy generation, etc.

The downstream applications of micro-nano optics are extensive, with public security anti-counterfeiting, laser packaging materials, and display and lighting being the three most representative industry applications. Meanwhile, the electronic device industry has entered the next generation of nanotechnology, in which optical super-resolution techniques, fabricated micro-nano structures, manipulation of surface equipartition excitations, and other micro-nano optical technologies and fast phase change materials have received extensive attention and research. In the field of photoelectric detection, artificial micro-nano structured photodetectors have surpassed traditional material detection performance in certain performance indicators. In particular, the artificial micro-nano structure-based photodetector shows excellent photoconversion performance at room temperature and will be one of the most competitive detector technologies for the next generation of high-performance, uncooled photodetection. For the bright application prospect, micro nano optics deserves more attention from researchers.
